# Relationship Between Age, Conditioning Intensity, and Outcome After Allografting in Adults Age ≥60 Years with AML

**DOI:** 10.21203/rs.3.rs-5220097/v1

**Published:** 2024-11-15

**Authors:** Phuong Vo, Brenda Sandmaier, Megan Othus, naveed ali, Eduardo Rodríguez-Arbolí, Corentin Orvain, Chris Davis, Ryan Basom, Rainer Storb, Roland Walter

**Affiliations:** Fred Hutchinson Cancer Research Center; Fred Hutchinson Cancer Center; Fred Hutchinson Cancer Center; Fred Hutchinson Cancer Center; Department of Hematology, Hospital Universitario Virgen del Rocío; Angers University Hospital; Fred Hutchinson Cancer Research Center; Fred Hutchinson Cancer Center; Fred Hutchinson Cancer Research Center / Univ. of Washington; Fred Hutchinson Cancer Center

## Abstract

Methodological advancements now allow older adults with AML to receive allografts although conflicting data exist regarding relative outcomes across age groups and benefits of different conditioning intensities. We retrospectively analyzed 495 adults aged 60–64 (n = 184), 65–69 (n = 189), or ≥ 70 (n = 122) who underwent allogeneic HCT for AML in remission at our institution from 2006 to 2023. There were no significant differences in relapse or relapse-free survival (RFS) among the 3 age cohorts after multivariable adjustment. Patients aged ≥ 70 years had a higher risk of non-relapse mortality (NRM) than those aged ≥ 60–64 (*P* = 0.022) but their overall survival (OS) was only statistically non-significantly shorter (*P* = 0.11). There was an important interplay between age, conditioning intensity, and outcomes. Age ≥ 70 years was associated with a higher risk of relapse (hazard ratio [HR] = 3.47; *P* = 0.012) and NRM (HR = 3.88; *P* = 0.001) with reduced intensity conditioning (RIC), leading to shorter RFS (HR = 3.79; *P* < 0.001) and OS (HR = 3.46; *P* < 0.001), while no association was found with nonmyeloablative conditioning. Conversely, patients aged 60–64 and 65–69, not those aged ≥ 70, had a significantly lower risk of relapse with RIC, but NRM risk increased with age. Our findings support allogeneic HCT for adults with AML in remission even if aged beyond 70, especially with nonmyeloablative conditioning.

## INTRODUCTION

With a median age of nearly 70 years at diagnosis, acute myeloid leukemia (AML) is a disease primarily affecting older adults.^1–4^ For most, allogeneic hematopoietic cell transplantation (HCT) remains the only curative therapy, but this approach comes with high rates of non-relapse mortality (NRM), particularly in those with comorbidities.^5–7^ The development of reduced intensity conditioning (RIC) and nonmyeloablative (NMA) conditioning regimens has expanded the use of allogeneic HCT to include older and medically less fit patients.^5–10^ Indeed, transplants performed in such adults with AML have increased over the last 2 decades. Nonetheless, only ~ 5% of those age > 70 receive allografts even today. Partly, that is because advanced age is still perceived as a barrier to allografting.^11–13^ Whether such concerns are justified in modern times is unclear. In a recent Center for International Blood and Marrow Transplant Research (CIBMTR) analysis of 1,321 adults age ≥ 60 years with AML in first remission, outcomes of patients age ≥ 70 were only slightly inferior to those aged 60–64, with age having only a small effect on overall survival (OS) and non-relapse mortality (NRM) after multivariable adjustment.^6^ In a study by the European Society for Blood and Marrow Transplantation (EBMT) of 701 adults age ≥ 70 with AML in first remission, similar outcomes were observed as those reported by the CIBMTR, but results were not contrasted to outcomes of patients younger than age 70.^7^ On the other hand, in a retrospective comparison of outcomes of 1,547 adults age 70–79 undergoing allogeneic HCT for a variety of neoplasms with those of 9,422 patients age 60–69, similar rates of relapse but higher NRM were found in the older patient cohort, translating into inferior OS and relapse-free survival (RFS).^9^ As these data were all derived from registry databases, we here retrospectively examined outcomes of 493 adults ≥ 60 years of age who underwent allogeneic HCT for AML in first or second remission between 2006 and 2023 at our institution, focusing on the relationship between age, conditioning intensity, and outcomes between individuals aged 60–64, aged 65–69, and those aged ≥ 70.

## PATIENTS AND METHODS

### Study cohort

We analyzed data on 495 patients ≥ 60 years with a diagnosis of AML or MDS/AML (2022 International Consensus Classification [ICC] criteria^14^) in first and second morphologic remission (i.e. <5% blasts in bone marrow) who underwent HCT at Fred Hutchinson Cancer Center from 05/2006 to 03/2023, agreed to their data being used for research purposes, and underwent institutional measurable residual disease (MRD) testing during the pre-HCT work-up. Related or unrelated donors were selected by high-resolution HLA-typing. Information on post-HCT outcomes was captured via the Long-Term Follow-Up Program through medical records from our outpatient clinic and local clinics that provided primary care for patients in addition to records obtained on patients on research studies. All patients were treated on Institutional Review Board-approved research protocols (all registered with ClinicalTrials.gov) or standard treatment protocols and gave consent in accordance with the Declaration of Helsinki. Follow-up was current as of July 30, 2024.

### Classification of comorbidity burden, disease risk, and treatment response

The HCT-specific comorbidity index (HCT-CI) was calculated as described.^15^ The 2022 European LeukemiaNet criteria^16^ were used to assign cytogenetic risk at diagnosis. Only the cytogenetic component of the risk classification could be used for risk assessment because molecular data at time of diagnosis were lacking in many patients. Secondary AML was defined using the 2022 ELN criteria.^16^ Treatment responses were categorized as proposed by the 2022 ELN criteria^16^ except that post-HCT relapse was defined as emergence > 5% blasts by morphology or multiparameter flow cytometry (MFC) in blood or bone marrow, emergence of cytogenetic abnormalities seen previously, or presence/emergence of any level of disease if leading to a therapeutic intervention.

### Types and intensity of conditioning regimens

High-dose fractionated total body irradiation (TBI ≥ 12Gy) with or without cyclophosphamide (CY) or fludarabine (FLU), high-dose TBI/thiotepa/FLU, busulfan (4 days) with CY or FLU, treosulfan/FLU with or without low-dose TBI, or any regimen containing a radiolabeled antibody were considered myeloablative conditioning (MAC) regimens. Reduced intensity conditioning (RIC) regimens included clofarabine with low dose TBI, melphalan (MEL)/FLU with or without low-dose TBI, CY/thiotepa/FLU/low dose TBI, busulfan (2 days)/FLU, FLU with 4–4.5 GY TBI and cladribine/cytarabine/G-CSF/mitoxantrone (CLAG-M) with low-dose TBI. FLU and low-dose TBI regimens were considered NMA regimens.

### MFC-based MRD testing

All patients underwent bone marrow aspirate analysis with ten-color flow cytometry as part of the pre-HCT work-up as described.^17^ Any detectable MRD was considered positive.^18^

### Statistical analysis

For our analyses, we considered 3 age groups: age 60–64, age 65–69, and age ≥ 70 comparing patient, disease, and HCT treatment characteristics. Unadjusted probabilities of RFS (events = relapse and death) and OS (event = death) were estimated using the Kaplan-Meier method, and probabilities of relapse and NRM were summarized using cumulative incidence estimates. NRM was defined as death without prior relapse and was considered a competing risk for relapse, while relapse was a competing risk for NRM. OS, RFS, NRM, and relapse were measured from date of transplant; patients last known to be alive without event were censored at date of last contact. Associations with RFS and OS were assessed using Cox regression; cause-specific regression models were used for relapse and NRM. Categorical patient characteristics were compared using Pearson’s Chi-squared (χ^2^) test and quantitative characteristics were compared with the Wilcoxon rank sum test. Two-sided *P*-values are reported. Statistical analyses were performed using R (http://www.r-project.org).

### Data sharing statement

For original, de-identified data, please contact the corresponding author (rwalter@fredhutch.org).

## RESULTS

### Characteristics of study cohort

We identified 495 adults with either AML (n = 418 [84%]) or MDS/AML (n = 77 [16%]) for inclusion in our analyses. Of these, 184 [37%] were age 60–64 years, 189 [38%] age 65–69 years, and 122 [25%] ≥ 70 years old, respectively. As summarized in Table 1, there were no statistically significant differences between these three groups regarding gender, ECOG performance status, disease type (AML *vs*. MDS/AML), cytogenetic risk, remission status at HCT (first *vs*. second remission), proportion of with patients with MRD by MFC pre-HCT, residual cytogenetic abnormalities at the time of HCT, HCT-CI score distribution, or stem cell source used for allografting. On the other hand, there were statistically significant differences in donor type/HLA disparity (*P* = 0.017) and more patients within the youngest age group receiving MAC (*P* < 0.001). Related to these differences, the type of graft-versus-host (GVHD) prophylaxis used differed between groups (*P* < 0.001; Table 1).

### Relationship between age group and post-HCT outcome

**Figure 1:**
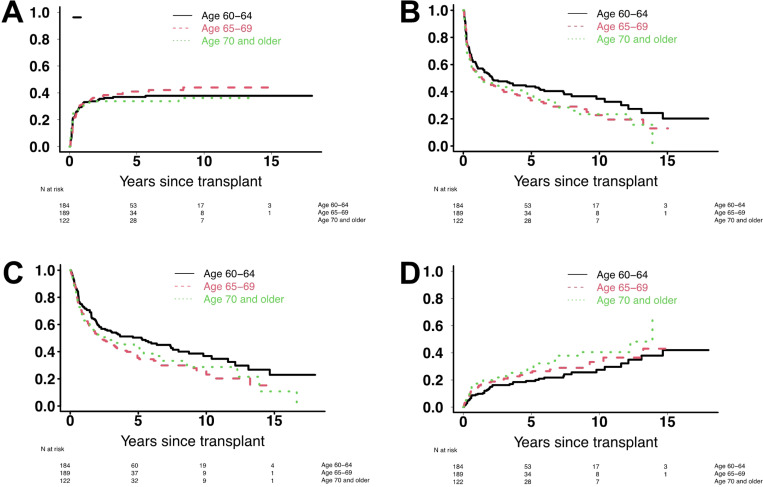
Post-HCT outcomes for 495 adults with AML or MDS/AML age ≥60 years undergoing allogeneic HCT while in first or second morphologic remission, stratified by age cohort. (**A**) Risk of relapse, (**B**) relapse-free survival, (**C**) overall survival, and (**D**) risk of non-relapse mortality, shown separately for patients aged 60–64 years (n=184), 65–69 years (n=189), and ≥70 years (n=122), respectively.

**Figure 2:**
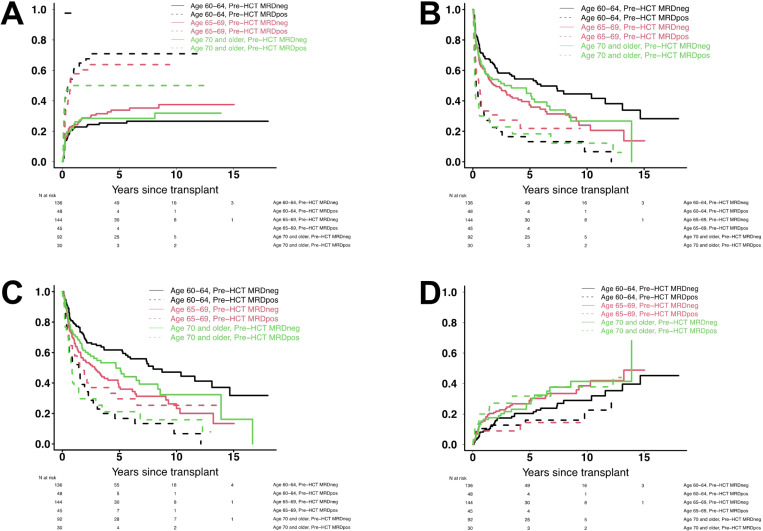
Post-HCT outcomes for 495 adults with AML or MDS/AML undergoing allogeneic HCT while in first or second morphologic remission, stratified by age cohort and pre-HCT MRD status. (**A**) Risk of relapse, (**B**) relapse-free survival, (**C**) overall survival, and (**D**) risk of non-relapse mortality, shown separately for MRD^neg^ and MRD^pos^ patients aged 60–64 years (n=136 and n=48), MRD^neg^ and MRD^pos^ patients aged 65–69 years (n=144 and n=45), and MRD^neg^ and MRD^pos^ patients aged ≥70 years (n=92 and n=30), respectively.

With a median follow-up of 51 (range: 6–204) months after HCT among survivors, there were 302 deaths, 186 relapses, and 135 NRM events contributing to the probability estimates for relapse, OS, RFS, and NRM in this cohort. In a first analysis, we assessed the relationship between age at the time of HCT (i.e. age 60–64 *vs*. age 65–69 *vs*. age ≥ 70) and post-HCT outcome in the entire study cohort. As summarized in Table 2 and depicted in Figs. 1 and 2, the risk of NRM increased in a stepwise fashion with advancing age, with a markedly higher risk among patients with pre-HCT MRD by MFC. On the other hand, relapse risks were similar across the three age groups. Three-year estimates of RFS and OS were higher for individuals aged 60–64 than those aged ≥ 65 years but similar between patients aged 65–69 and those aged ≥ 70 (for RFS: 48% *vs*. 40% *vs*. 42%; for OS: 54% *vs*. 45% *vs*. 47%).

### Age group as independent prognostic factor for post-HCT outcomes

To study the relationship between age and post-HCT outcomes in more detail, we evaluated univariate and multivariable regression models for the risk of relapse, risk of relapse/death, risk of death, and risk of NRM. In univariate analyses, there were no statistically significant differences in the risk of relapse, risk of relapse/death, and risk of death between patients aged 60–64, 65–69, and ≥ 70 years (Supplemental Table 1). In contrast, the risk of NRM was statistically higher in patients ≥ 70 years of age relative to those age 60–64 (*P* = 0.017), whereas patients aged 65–69 had a non-significantly higher risk of NRM relative to patient in the youngest age group (*P* = 0.12).

In addition to age, several other patient-, disease-, and HCT-related characteristics were associated with either relapse, RFS, OS, and/or NRM, including gender, performance status, HCT-CI score, disease type, cytogenetic disease risk, remission number, pre-HCT MRD status by MFC, residual cytogenetic abnormalities, type of donor/HLA disparity, conditioning intensity, and type of GVHD prophylaxis (Supplemental Table 1). After multivariable adjustment, patients aged ≥ 70 years had a statistically significantly higher risk of NRM risk than those age ≥ 60–64 (hazard ratio [HR] = 1.75 [95% confidence interval: 1.08–2.82]; *P* = 0.022), and their risk of death was slightly but statistically non-significantly higher (HR = 1.29 [0.94–1.78]; *P* = 0.11), whereas risks of relapse and relapse/death were not significantly different (Table 3). There were no statistically significant differences regarding relapse, RFS, OS, or NRM between patients aged 60–64 years and those aged 65–69 years after multivariable adjustment. On the other hand, a poor performance status (ECOG 2–3) was associated with statistically significantly higher risk of relapse (HR = 2.53 [1.56–4.11]; *P* < 0.001), shorter RFS (HR = 2.75 [1.86–4.05]; *P* < 0.001), shorter OS (HR = 2.67 [1.79–3.99]; *P* < 0.001), and higher NRM (HR = 3.37 [1.75–6.50]; *P* < 0.001). There was also a statistically significant association between HCT-CI score ≥ 4 and increased NRM (HR = 1.98 [1.26–3.12]; *P* = 0.0031) and OS (HR = 1.40 [1.04–1.88]; *P* = 0.026) but not relapse (*P* = 0.89) or RFS (*P* = 0.12), after adjustment.

### Age group as independent prognostic factor for post-HCT outcomes in distinct patient subsets

Because our study cohort was heterogeneous not only with regard to patient age but also other patient and donor characteristics, we examined the relationship between age and post-HCT outcomes in subset analyses in which we restricted our analyses separately to: the 416 patients with AML, the 369 patients who received an allograft from a 10/10 HLA-identical/matched related or unrelated donor, the 371 patients who tested negative for MFC MRD pre-HCT, the 122 patients who tested positive for MFC MRD pre-HCT, the 240 patients who received nonmyeloablative conditioning, and the 144 patients who received RIC before allografting (Table 4). After multivariable adjustment, findings in the patients with AML and those who received an allograft from a 10/10 HLA-identical/matched related or unrelated donor were like those obtained in the entire study cohort. In the subset of patients receiving NMA conditioning, there was no statistically significant association between age group and relapse, RFS, OS, or NRM. In contrast, among patients allografted after RIC, age ≥ 70 years was associated with higher risk of relapse (HR = 3.29 [1.22–8.86]; *P* = 0.018, shorter RFS (HR = 3.47 [1.85–6.65]; *P* < 0.001), shorter OS (HR = 3.14 [1.64–6.02]; *P* = 0.001), and higher risk of NRM (HR = 3.78 [1.65–8.67]; *P* = 0.002).

### Interplay between age group, conditioning intensity and post-HCT outcomes

The above findings suggested an interaction between age group, conditioning intensity, and outcomes after allografting. To investigate this relationship further, we tested the interaction and when found it was significant, we built multivariable models for each age group and assessed the relative risks of relapse, relapse/death, death, and NRM for patients receiving RIC relative to those receiving NMA conditioning. After adjustment for gender, HCT-CI score, disease type, disease risk (adverse vs. non-adverse, due to small patient subsets), remission status, pre-HCT MRD, and residual cytogenetic abnormalities at HCT, patients aged 60–64 and 65–69 but not those aged ≥ 70 had a significantly lower risk of relapse when receiving RIC. On the other hand, whereas the risk of NRM was similar for those receiving NMA conditioning and those receiving RIC in the 60–64-year age group, the relative NRM risk increased with age in stepwise fashion with RIC, with patients ≥ 70 years of age having a markedly increased of NRM when receiving RIC rather than NMA conditioning (HR = 6.60 [2.72–16.00]; *P* < 0.001). This resulted in a statistically significantly better RFS (HR = 0.49 [0.29–0.85]; *P* = 0.010) with RIC among patients aged 60–64, whereas RIC was associated with worse RFS (HR = 2.15 [1.24–3.75]; *P* = 0.007) and OS (HR = 2.73 [1.52–4.92]; *P* = 0.001) in patients aged ≥ 70 relative to those receiving NMA conditioning (Table 4).

## DISCUSSION

In our cohort, almost a quarter of adults undergoing allogeneic HCT for AML in first or second remission were ≥ 70 years old – a testament as to how advances in transplant platforms and supportive care have enabled allografting in patients of advanced age. Retrospectively comparing their outcomes to those of patients aged 60–64 or 65–69, our data allow 2 main conclusions: first, there were no statistically significant differences in relapse or RFS between the 3 age cohorts. In contrast, patients aged ≥ 70 years had a higher risk of NRM than those aged ≥ 60–64 and their OS was slightly but statistically non-significantly shorter. And second, there was an important interplay between age, conditioning intensity, and post-HCT outcomes. Specifically, while there was no association between age group and relapse, RFS, OS, or NRM in patients receiving NMA conditioning, age ≥ 70 years was associated with higher risk of relapse and NRM and, consequently, shorter RFS and OS in those receiving RIC. Underlying this interplay, we found patients aged 60–64 and 65–69 but not those aged ≥ 70 had a significantly lower risk of relapse when receiving RIC rather than NMA conditioning, whereas the relative NRM risk increased with age in stepwise fashion with RIC compared to NMA conditioning. Together, our findings support use of allogeneic HCT for adults aged ≥ 70 with AML in remission, especially following NMA conditioning.

In the recent CIBMTR study,^6^ adults aged 60–64 receiving an allograft for AML in first remission experienced better OS and RFS and lower TRM than those aged 65–69 or aged ≥ 70, but the difference in survival was relatively modest (at 3 years: 49% *vs*. 42% *vs*. 45%). Our findings differ slightly from this large registry analysis in that, in our cohort, we found outcomes for patients aged 60–64 to be like those for patients aged 65–69, and we could only find a statistically significant worsening in NRM risk but not survival outcomes for those aged ≥ 70. Still, these two studies are congruent in that they indicate age ≥ 70 years alone should not be considered a barrier to allogeneic HCT and should not exclude patients from this treatment strategy.

In line with data from the recent CIBMTR analysis,^6^ a higher HCT-CI score was associated with increased NRM risk and worse survival estimates in our study cohort. These findings highlight the importance of co-morbid illnesses as factor impacting post-HCT outcomes, suggesting the need to centrally consider measures of biological rather than just chronological age in the decision-making process regarding allogeneic HCT in older adults.

For younger adults with AML, some data have indicated better outcomes with MAC *vs*. RIC.^19,20^ However, with advancing age and/or presence of comorbid illnesses, tolerance for MAC decreases, and only a small proportion of adults ≥ 70 will receive MAC in preparation of allografting. Thus, most of these patients will receive either RIC or NMA conditioning, with an ongoing debate about their relative merits. In a prospective randomized phase 2 study of 139 younger adults (median age: 54 years) with various hematologic malignancies comparing RIC vs NMA conditioning, identical overall survival rates were observed, with the RIC group experiencing a lower rates of relapse that were counterbalanced by an increase in NRM.^21^ In a retrospective analysis of the European Society for Blood and Marrow Transplantation (EMBT) of 1,088 adults age ≥ 60, no differences were observed with respect to risk of relapse, RFS, OS, or NRM between those receiving a RIC regimen and those receiving NMA conditioning.^22^ Likewise, in a recent retrospective analysis of adults of all ages undergoing allografting for AML in first or second remission at our center, we found no significant differences in outcomes between those assigned to RIC and those assigned to NMA conditioning.^23^ Our present analysis points at an age group-dependent relative risk/benefit for RIC *vs*. NMA conditioning in older adults with AML. Specifically, for patients aged 60–64, RIC was associated with reduced relapse risk but no increase in NRM and, consequently, improved RFS and OS relative to NMA conditioning. In contrast, in patients aged ≥ 70, RIC was associated with substantially higher NRM and worse RFS and OS than NMA conditioning while the relapse risk was similar. Thus, patients aged ≥ 70 not only experienced more life-limiting toxicities with RIC but also did not benefit from a reduction in relapse risks with a more intensive conditioning strategy. This observation may serve as a basis for advocating the routine utilization of NMA conditioning modalities in patients aged ≥ 70. Uncertainty remains for the group of patients aged 65–69, in whom RIC was associated with a reduced risk of relapse but an increase in NRM and, hence, comparable RFS and OS relative to NMA conditioning. Further investigations are needed to optimize outcomes in these individuals by identifying most ideal conditioning regimen(s).

As strengths, ours was a study encompassing a relatively large number of older adults allografted at a single institution with use of uniform and consistent, standardized supportive care. On the other hand, several limitations must be acknowledged. First, this is a retrospective analysis of patients who were assigned to different conditioning and immune suppressive regimens largely in a non-randomized fashion. Second, while the NMA conditioning regimen was uniform, several types of MAC and RIC regimens were used, and it is possible relative risks/benefits differ between individual regimens. Because of the limited cohort sizes for many of the regimens used, this possibility could not be studied rigorously. And third, no uniform treatment strategies were used for patients who tested positive for MRD in the post-HCT period. Therapies were selected largely based on preference of the clinical HCT team, and included expedited withdrawal of immunosuppressive agents, infusion of donor lymphocytes, treatment with hypomethylating agents or molecularly targeted agents, either alone or in various simultaneous or consecutive combinations. Acknowledging these limitations, our findings support use of allogeneic HCT for older adults beyond age 70 with AML in remission. While needing independent validation, our data point to NMA conditioning as the preferred strategy for allogeneic HCT in adults aged ≥ 70 given relapse rates are like those after RIC, but NRM rates are significantly lower, translating into better survival estimates with NMA conditioning than RIC in adults of very advanced age.

## Supplementary Material

Supplement 1

## Figures and Tables

**Table 1:** Pre-HCT demographic and clinical characteristics of study population

	All patients (n = 493)	Age 60–64 (n = 184)	Age 65–69 (n = 189)	Age ≥ 70 (n = 120)	*P*-value
**Median age at HCT (range), years**	66.7 (60.1–80.9)	62.5 (60.1–65.0)	67.6 (65.0–69.9)	72.5 (70.0–80.9)	0.0001

**Female gender, n (%)**	204 (41)	80 (43)	77 (41)	47 (39)	0.69

**ECOG performance status, n (%)**	456 (92)	172 (91)	172 (91)	112 (92)	0.63
0–1	37 (7)	12 (7)	17 (9)	8 (7)	
2–3	2 (< 1)	0 (0)	0 (0)	2 (2)	
Missing					

**HCT Comorbidity Index, n (%)**	180 (36)	76 (41)	64 (34)	40 (33)	0.15
0–1	157 (32)	46 (55)	66 (35)	45 (37)	
2–3	156 (32)	62 (34)	59 (31)	35 (29)	
≥4	2 (< 1)	0 (0)	0 (0)	2 (2)	
Missing					

**Disease, n (%)**	418 (84)	151 (82)	158 (84)	109 (89)	0.29
AML	290 (59)	110 (60)	104 (55)	76 (62)	
*De novo* AML	128 (26)	41 (22)	54 (29)	33 (27)	
Secondary AML	77 (16)	33 (18)	31 (16)	13 (11)	
MDS/AML					

**Cytogenetic risk at diagnosis, n (%)**	9 (2)	2 (1)	2 (1)	5 (4)	0.55
Favorable	320 (65)	124 (68)	119 (63)	75 (63)	
Intermediate	148 (30)	52 (28)	60 (32)	36 (30)	
Adverse	18 (4)	6 (3)	8 (4)	4 (3)	
Missing					

**Remission status at HCT, n (%)**	419 (85)	157 (85)	157 (83)	105 (86)	0.75
First remission	76 (15)	27 (15)	32 (17)	17 (14)	
Second remission					

**Pre-HCT MRD status by flow cytometry, n (%)**	371 (75)	136 (74)	144 (76)	92 (75)	0.87
MRD^neg^	122 (25)	48 (26)	45 (24)	30 (25)	
MRD^pos^					

**Cytogenetics before HCT, n (%)**	150 (30)	47 (26)	57 (30)	46 (38)	0.097
Normalized karyotype	132 (27)	45 (24)	55 (29)	32 (26)	
Abnormal karyotype	213 (43)	92 (50)	77 (41)	44 (36)	
Non-informative data*					

**HLA matching, n (%)**	91 (18)	45 (24)	35 (19)	11 (9)	0.017
10/10 HLA-identical related donor	280 (57)	94 (51)	105 (56)	81 (66)	
10/10 HLA-matched unrelated donor	66 (13)	22 (12)	26 (14)	18 (15)	
1–2 allele/antigen mismatched unrelated donor	27 (5)	8 (4)	10 (5)	9 (7)	
HLA-haploidentical donor	31 (6)	15 (8)	13 (7)	3 (2)	
Umbilical cord blood					

**Source of stem cells, n (%)**	450 (91)	161 (88)	171 (90)	118 (97)	0.095
Peripheral blood	14 (3)	8 (4)	5 (3)	1 (1)	
Bone marrow	31 (6)	15 (8)	13 (7)	3 (2)	
Umbilical cord blood					

**Conditioning intensity, n (%)**	109 (22)	67 (36)	31 (16)	11 (9)	< 0.001
Myeloablative	144 (29)	52 (28)	64 (34)	28 (23)	
Reduced intensity	242 (49)	65 (35)	94 (50)	83 (68)	
Nonmyeloablative					

**GVHD prophylaxis, n (%)**	346 (70)	108 (59)	139 (74)	99 (81)	< 0.001
CNI + MMF ± sirolimus	57 (12)	43 (23)	14 (7)	0 (0)	
CNI + MTX ± other	91 (18)	33 (18)	35 (19)	23 (19)	
PTCy	1 (< 1)	0 (0)	1 (1)	0 (0)	
Other					

* Normal cytogenetics in patient with cytogenetically normal AML or missing cytogenetics at diagnosis.

** ANC ≥ 1,000/μL and platelets ≥ 100,000/μL. Abbreviations: AML, acute myeloid leukemia; CNI, calcineurin inhibitor; GVHD, graft-versus-host disease; HCT, hematopoietic cell transplantation; HLA, human leukocyte antigen; MDS, myelodysplastic neoplasm; MMF, mycophenolate mofetil; MRD, measurable residual disease; MTX, methotrexate; PTCy, post-transplantation cyclophosphamide.

**Table 2:** Outcome probabilities (with 95% confidence interval) stratified by age group and pre-HCT MRD status

	Relapse at 3 years	RFS at 3 years	OS at 3 years	NRM at 100 days	NRM at 3 years
**All patients** (n = 493)	36% (32–41%)	44% (39–48%)	50% (45–55%)	5% (3–7%)	20% (16–23%)

Pre-HCT MRD status	28% (24–33%)	51% (46–56%)	56% (52–62%)	5% (3–7%)	21% (17–26%)
MRD^neg^ (n = 371)	63% (54–71%)	22% (16–31%)	30% (22–40%)	7% (3–12%)	15% (9–22%)
MRD^pos^ (n = 122)					

**Age 60–64** (n = 184)	36% (29–43%)	48% (41–56%)	55% (48–63%)	3% (1–6%)	16% (11–22%)

Pre-HCT MRD status	24% (18–32%)	58% (50–67%)	65% (57–73%)	3% (1–7%)	17% (11–24%)
MRD^neg^ (n = 136)	71% (55–82%)	16% (8–33%)	26% (15–43%)	4% (1–10%)	13% (5–24%)
MRD^pos^ (n = 48)					

**Age 65–69** (n = 189)	39% (32–46%)	40% (33–48%)	45% (39–53%)	6% (4–10%)	22% (15–27%)

Pre-HCT MRD status	32% (24–39%)	44% (36–53%)	48% (40–57%)	6% (3–11%)	25% (18–32%)
MRD^neg^ (n = 144)	64% (47–77%)	27% (17–45%)	37% (25–55%)	7% (2–17%)	9% (3–20%)
MRD^pos^ (n = 45)					

**Age ≥ 70** (n = 120)	34% (26–42%)	43% (35–53%)	49% (40–59%)	7% (3–12%)	23% (16–31%)

Pre-HCT MRD status	29% (20–38%)	50% (41–62%)	56% (47–68%)	4% (1–10%)	22% (14–31%)
MRD^neg^ (n = 91)	50% (31–67%)	23% (12–44%)	25% (14–48%)	13% (4–28%)	27% (12–44%)
MRD^pos^ (n = 29)					

Abbreviations: HCT, hematopoietic cell transplantation; MRD, measurable residual disease; NRM, non-relapse mortality; OS, overall survival; RFS, relapse-free survival

**Table 3:** Multivariable regression models (entire study cohort)

	Risk of relapse		Risk of relapse/death		Risk of death		Risk of NRM	
	HR (95% CI)	*P*-value	HR (95% CI)	*P*-value	HR (95% CI)	*P*-value	HR (95% CI)	*P*-value
**Age group (ref: 60–64 years)**	1.13 (0.80–1.59)	0.40	1.15 (0.89–1.49)	0.29	1.22 (0.93–1.60)	0.15	1.17 (0.77–1.79)	0.47
65–69 years	1.13 (0.74–1.71)	0.57	1.34 (0.98–1.82)	0.062	1.29 (0.94–1.78)	0.11	1.75 (1.08–2.82)	0.022
≥70 years								

**Male gender (ref: female gender)**	0.82 (0.61–1.11)	0.20	1.11 (0.88–1.40)	0.39	1.28 (1.01–1.63)	0.043	1.78 (1.22–2.61)	0.003

**ECOG performance status (ref: 0–1)**	2.53 (1.56–4.11)	<0.001	2.75 (1.86–4.05)	<0.001	2.67 (1.79–3.99)	<0.001	3.37 (1.75–6.50)	<0.001
2–3								

**HCT Comorbidity Index (ref: 0–1)**	0.97 (0.67–1.41)	0.88	1.06 (0.80–1.40)	0.71	1.09 (0.81–1.46)	0.56	1.31 (0.84–2.06)	0.24
2–3	0.97 (0.66–1.43)	0.89	1.26 (0.94–1.67)	0.12	1.40 (1.04–1.88)	0.026	1.98 (1.26–3.12)	0.0031
≥4								

**Disease type (ref: de novo AML)**	1.25 (0.88–1.77)	0.21	1.15 (0.88–1.49)	0.32	0.97 (0.74–1.28)	0.84	1.10 (0.73–1.67)	0.64
Secondary AML	1.15 (0.73–1.82)	0.55	1.26 (0.89–1.77)	0.19	1.38 (0.98–1.95)	0.068	1.42 (0.84–2.42)	0.19
MDS/AML								

**Cytogenetic risk (ref: intermediate)**	0.20 (0.03–1.53)	012	0.46 (0.19–1.14)	0.092	0.49 (0.19–1.28)	0.15	0.55 (0.19–1.57)	0.26
Favorable	2.54 (1.71–3.77)	<0.001	1.58 (1.18–2.13)	0.0025	1.39 (1.02–1.89)	0.037	0.67 (0.41–1.10)	0.11
Adverse								

**Disease status (ref: first remission)**	1.85 (1.21–2.82)	0.0043	1.65 (1.18–2.30)	0.0032	1.41 (1.01–1.97)	0.045	1.47 (0.85–2.56)	0.17
Second remission								

**Pre-HCT MRD status (ref: MRD^neg^)**	2.58 (1.85–3.60)	<0.001	1.95 (1.49–2.54)	<0.001	1.58 (1.20–2.08)	0.0011	1.14 (0.71–1.85)	0.59
MRD^pos^								

**Pre-HCT karyotype (ref: normalized)**	1.34 (0.91–1.96)	0.14	1.28 (0.95–1.74)	0.10	1.40 (1.02–1.92)	0.036	1.15 (0.69–1.93)	0.59
Not normalized								

**HLA donors (ref: HLA-identical relative)**	0.86 (0.57–1.28)	0.45	0.84 (0.61–1.15)	0.27	0.94 (0.68–1.29)	0.69	0.86 (0.52–1.42)	0.55
10/10 HLA-matched unrelated donor	0.60 (0.33–1.07)	0.084	0.92 (0.61–1.39)	0.68	1.10 (0.72–1.69)	0.65	1.42 (0.76–2.65)	0.27
1–2 allele/antigen mismatched unrelated donor	1.07 (0.56–2.04)	0.85	1.08 (0.62–1.87)	0.79	1.13 (0.64–1.98)	0.68	1.13 (0.40–3.16)	0.82
HLA-haploidentical donor	1.04 (0.53–2.04)	0.90	1.60 (0.98–2.61)	0.061	1.58 (0.94–2.65)	0.082	2.64 (1.27–5.50)	0.0097
Umbilical cord blood								

**Conditioning (ref: nonmyeloablative)**	0.52 (0.35–0.78)	0.0014	0.84 (0.63–1.12)	0.23	0.92 (0.69–1.23)	0.56	1.54 (1.02–2.34)	0.042
Reduced intensity	0.71 (0.48–1.05)	0.089	0.75 (0.54–1.02)	0.07	0.82 (0.59–1.15)	0.26	0.74 (0.42–1.28)	0.28
Myeloablative								

Abbreviations: AML, acute myeloid leukemia; HCT, hematopoietic cell transplantation; HLA, human leukocyte antigen; MDS, myelodysplastic neoplasm; MRD, measurable residual disease.

**Table 4:** Multivariable regression models for distinct patient subsets

	Risk of relapse		Risk of relapse/death		Risk of death		Risk of NRM	
	HR (95% CI)	*P*-value	HR (95% CI)	*P*-value	HR (95% CI)	*P*-value	HR (95% CI)	*P*-value
**AML patients only (n = 418)**								

**Age group (ref: 60–64 years)**	1.49 (1.02–2.17)	0.037	1.28 (0.96–1.71)	0.09	1.26 (0.93–1.70)	0.13	0.95 (0.60–1.52)	0.83
65–69 years	1.17 (0.74–1.84)	0.51	1.30 (0.93–1.80)	0.12	1.24 (0.88–1.75)	0.22	1.52 (0.93–2.48)	0.10
≥70 years								

**Patients receiving 10/10 HLA-identical/matched related or unrelated donor allografts only (n = 371)**								

**Age group (ref: 60–64 years)**	1.25 (0.85–1.85)	0.26	1.28 (0.94–1.75)	0.12	1.42 (1.03–1.96)	0.035	1.25 (0.74–2.14)	0.39
65–69 years	1.11 (0.68–1.81)	0.67	1.50 (1.04–2.15)	0.031	1.52 (1.03–2.24)	0.033	2.49 (1.40–4.43)	0.002
≥70 years								

**Patients without MFC MRD pre-HCT (n = 371)**								

**Age group (ref: 60–64 years)**	1.14 (0.72–1.83)	0.56	1.17 (0.84–1.64)	0.35	1.41 (0.99–1.98)	0.052	1.21 (0.76–1.93)	0.43
65–69 years	1.10 (0.63–1.88)	0.75	1.27 (0.86–1.85)	0.22	1.34 (0.91–2.01)	0.14	1.50 (0.88–2.57)	0.14
≥70 years								

**Patients with MFC MRD pre-HCT (n = 122)**								

**Age group (ref: 60–64 years)**	1.08 (0.63–1.87)	0.78	0.99 (0.61–1.62)	0.98	0.80 (0.48–1.34)	0.40	0.47 (0.13–1.72)	0.26
65–69 years	1.60 (0.77–3.33)	0.21	1.77 (0.97–3.22)	0.062	1.39 (0.74–2.61)	0.31	1.90 (0.60–6.21)	0.29
≥70 years								

**Patients receiving nonmyeloablative conditioning (n = 242)**								

**Age group (ref: 60–64 years)**	1.21 (0.74–2.01)	0.44	1.21 (0.82–1.79)	0.34	1.22 (0.83–1.81)	0.31	1.04 (0.55–1.95)	0.90
65–69 years	0.95 (0.55–1.62)	0.85	0.94 (0.63–1.41)	0.77	0.89 (0.59–1.35)	0.58	0.85 (0.45–1.60)	0.63
≥70 years								

**Patients receiving reduced intensity conditioning (n = 144)**								

**Age group (ref: 60–64 years)**	1.56 (0.70–3.68)	0.25	1.37 (0.79–2.41)	0.26	1.41 (0.77–2.58)	0.26	1.14 (0.51–2.52)	0.5
65–69 years	3.29 (1.22–8.86)	0.018	3.47 (1.85–6.65)	0.001	3.14 (1.64–6.02)	0.001	3.78 (1.65–8.67)	0.021
≥70 years								

**Patients aged 60–64 years (n = 184)**								

**Conditioning (ref: nonmyeloablative)**	0.31 (0.15–0.69)	0.004	0.49 (0.29–0.85)	0.010	0.60 (0.34–1.05)	0.07	0.78 (0.36–1.71)	0.54
Reduced intensity								

**Patients aged 65–69 years (n = 189)**								

**Conditioning (ref: nonmyeloablative)**	0.41 (0.23–0.75)	0.004	0.62 (0.41–0.95)	0.029	0.74 (0.48–1.13)	0.16	1.06 (0.56–2.02)	0.86
Reduced intensity								

**Patients aged ≥ 70 years (n = 122)**								

**Conditioning (ref: nonmyeloablative)**	0.93 (0.41–2.12)	0.86	2.15 (1.24–3.75)	0.007	2.73 (1.52–4.92)	<0.001	6.60 (2.72–16.00)	<0.001
Reduced intensity								

Abbreviations: AML, acute myeloid leukemia; MFC, multiparameter flow cytometry; MRD, measurable residual disease.

## Data Availability

For original, de-identified data, please contact the corresponding author (rwalter@fredhutch.org).
