# Supplementary material for: Relationship Between Age, Conditioning Intensity, and Outcome After Allografting in Adults Age ≥60 Years with AML
Source: Res Sq. 2024 Nov 15:rs.3.rs-5220097. Preprint. [Version 1] doi: 10.21203/rs.3.rs-5220097/v1 (PMC11601824; doi:10.21203/rs.3.rs-5220097/v1)
Supplement: Supplement 1 [file NIHPPRS5220097V1-supplement-1.pdf]

Supplementary Files

This is a list of supplementary files associated with this preprint. Click to download.

- LeukemiaAMLHCTinOlderAdultsDataSupplement20241007FINAL.docx
